# Acute impact of an endurance race on biventricular and biatrial myocardial strain in competitive male and female triathletes evaluated by feature-tracking CMR

**DOI:** 10.1007/s00330-021-08401-y

**Published:** 2021-12-13

**Authors:** Hang Chen, Malte L. Warncke, Kai Muellerleile, Dennis Saering, Antonia Beitzen-Heineke, Anna Kisters, Monika Swiderska, Ersin Cavus, Charlotte M. Jahnke, Gerhard Adam, Gunnar K. Lund, Enver Tahir

**Affiliations:** 1 Department of Diagnostic and Interventional Radiology and Nuclear Medicine, University Hospital Hamburg Eppendorf, Martinistr. 52, 20246 Hamburg, Germany; 2 Department of General and Interventional Cardiology, University Heart Center, Hamburg, Germany; 3 Information Technology and Image Processing, University of Applied Sciences, Wedel, Germany; 4 Department of Oncology, Hematology, BMT With Department of Pneumology, University Medical Center Hamburg, Hamburg, Germany

**Keywords:** Cardiac, Magnetic resonance imaging, Cine, Cardiac imaging techniques, Athletes

## Abstract

**Objectives:**

Cardiac adaptation in endurance athletes is a well-known phenomenon, but the acute impact of strenuous exercise is rarely reported on. The aim of this study was to analyze the alterations in biventricular and biatrial function in triathletes after an endurance race using novel feature-tracking cardiac magnetic resonance (FT-CMR).

**Methods:**

Fifty consecutive triathletes (45 ± 10 years; 80% men) and twenty-eight controls were prospectively recruited, and underwent 1.5-T CMR. Biventricular and biatrial volumes, left ventricular ejection fraction (LVEF), FT-CMR analysis, and late gadolinium imaging (LGE) were performed. Global systolic longitudinal (GLS), circumferential (GCS), and radial strain (GRS) were assessed. CMR was performed at baseline and following an endurance race. High-sensitive troponin T and NT-proBNP were determined. The time interval between race completion and CMR was 2.3 ± 1.1 h (range 1–5 h).

**Results:**

Post-race troponin T (*p* < 0.0001) and NT-proBNP (*p* < 0.0001) were elevated. LVEF remained constant (62 ± 6 vs. 63 ± 7%, *p* = 0.607). Post-race LV GLS decreased by tendency (− 18 ± 2 vs. − 17 ± 2%, *p* = 0.054), whereas GCS (− 16 ± 4 vs. − 18 ± 4%, *p* < 0.05) and GRS increased (39 ± 11 vs. 44 ± 11%, *p* < 0.01). Post-race right ventricular GLS (− 19 ± 3 vs. − 19 ± 3%, *p* = 0.668) remained constant and GCS increased (− 7 ± 2 vs. − 8 ± 3%, *p* < 0.001). Post-race left atrial GLS (30 ± 8 vs. 24 ± 6%, *p* < 0.0001) decreased while right atrial GLS remained constant (25 ± 6 vs. 24 ± 6%, *p* = 0.519).

**Conclusions:**

The different alterations of post-race biventricular and biatrial strain might constitute an intrinsic compensatory mechanism following an acute bout of endurance exercise. The combined use of strain parameters may allow a better characterization of ventricular and atrial function in endurance athletes.

**Key Points:**

• *Triathletes demonstrate a decrease of LV global longitudinal strain by tendency and constant RV global longitudinal strain following an endurance race*.

• *Post-race LV and RV global circumferential and radial strains increase, possibly indicating a compensatory mechanism after an acute endurance exercise bout*.

• *Subgroup analyses of male triathletes with focal myocardial fibrosis did not demonstrate alterations in biventricular and biatrial strain after an endurance race*.

**Supplementary Information:**

The online version contains supplementary material available at 10.1007/s00330-021-08401-y.

## Introduction

Numerous studies have reported on cardiac adaptations in endurance athletes [1–3], yet standard parameters of systolic function such as left ventricular ejection fraction (LVEF) might not detect subtle functional alterations of the athlete’s heart. Feature-tracking cardiac magnetic resonance (FT-CMR) imaging is an advanced technique for quantification of myocardial strain using conventional cine images [4]. Myocardial strain is defined as the relative change in fiber length from end diastole and can be a sensitive measure of myocardial deformation [5].

While normal values of ventricular and atrial strain in endurance athletes at rest are largely undefined, echocardiographic and CMR studies report that prolonged endurance training is associated with attenuated myocardial strain compared with sedentary controls, whereas biventricular ejection fractions are similar [6–10]. However, the acute changes of myocardial strain in response to an endurance race are less well studied. Previous echocardiographic studies have described differences in myocardial strain before and after a high-intensity exercise [11, 12]. Further, Christou et al showed that the absolute values of both ventricular longitudinal strains decreased after a race, but were still within normal range for the vast majority of athletes [13].

We hypothesized that triathletes develop acute cardiac alterations after an endurance race, which are directly or indirectly reflected by changes in myocardial function and increased cardiac biomarkers. The aim of this study was to analyze the alterations in biventricular and biatrial function in triathletes after an endurance race using novel FT-CMR.

## Methods and materials

### Triathletes and controls

The local ethics committee approved the study and all participants gave written informed consent. Triathletes were contacted through advertisements at triathlon clubs and were included if a minimum of 10 h weekly training and regular participation in official competitions in the last 3 years were given [14]. Control subjects were eligible with a weekly exercise of less than 3 h [14]. Study exclusion criteria were CMR contraindications, systemic disease, or cardiovascular diseases. No intake of any cardiac or illicit medication was reported [14]. Fifty consecutive male (80%; age: 44 ± 9 years, range 18–61 years) and female triathletes (age: 46 ± 11 years, range 29–62 years) underwent baseline and post-race CMR. Baseline CMR was acquired at least 30 days after the last race and subjects were instructed to refrain from any exercise in the preceding 72 h [14]. The interval between baseline and post-race CMR was more than a month. Baseline and post-race CMR findings of this cohort were partly reported previously and details are provided in the Supplementary Material [14–16]. This study expands the previous post-race cohort by nine male and eleven female triathletes. Blood samples were drawn immediately before each CMR from an antecubital vein in supine position for 5 min to obtain hematocrit, creatine kinase, high-sensitive troponin T, and N-terminal pro-brain natriuretic peptide (NT-proBNP) [14].

### Post-race CMR

All triathletes successfully finished their endurance races with a total cumulative race distance of 58 ± 62 km. Details on the endurance races are provided in the Supplementary Material. Post-race CMR was performed at 2.3 ± 1.1 h (range 1–5 h).

### CMR protocol

Studies were performed on a 1.5-T Achieva scanner with a 5-channel cardiac phased array receiver coil (Phillips, Healthcare). ECG-triggered steady-state free-precession (SSFP) cine sequences were acquired in short axis and 2-, 3-, and 4-chamber views. Native T1 mapping was performed using a 5 s(3 s)3 s modified look-locker inversion recovery (MOLLI) sequence on three short-axes slices [14]. A gradient (echo planar imaging) and spin-echo multi-echo sequence was applied to acquire native T2 mapping images in three short-axis slices. Ten minutes after injection of 0.15 mmol/kg gadoterate meglumine (Dotarem®, Guerbet), end-diastolic late gadolinium enhancement (LGE) images were acquired using end-diastolic phase-sensitive inversion recovery (PSIR) sequences in short-axis and 2-, 3-, and 4-chamber views. Additional details are given in the Supplementary Material.

### CMR data analysis

Two investigators (H.C. and M.W.) independently and blindly analyzed each CMR in random order using a commercially available software (CVi42, Circle Cardiovascular Imaging Inc.). CMR parameters were indexed to the body surface area (BSA). Additional details are provided in the Supplementary Material.

### Myocardial strain analysis

**Fig. 1:**
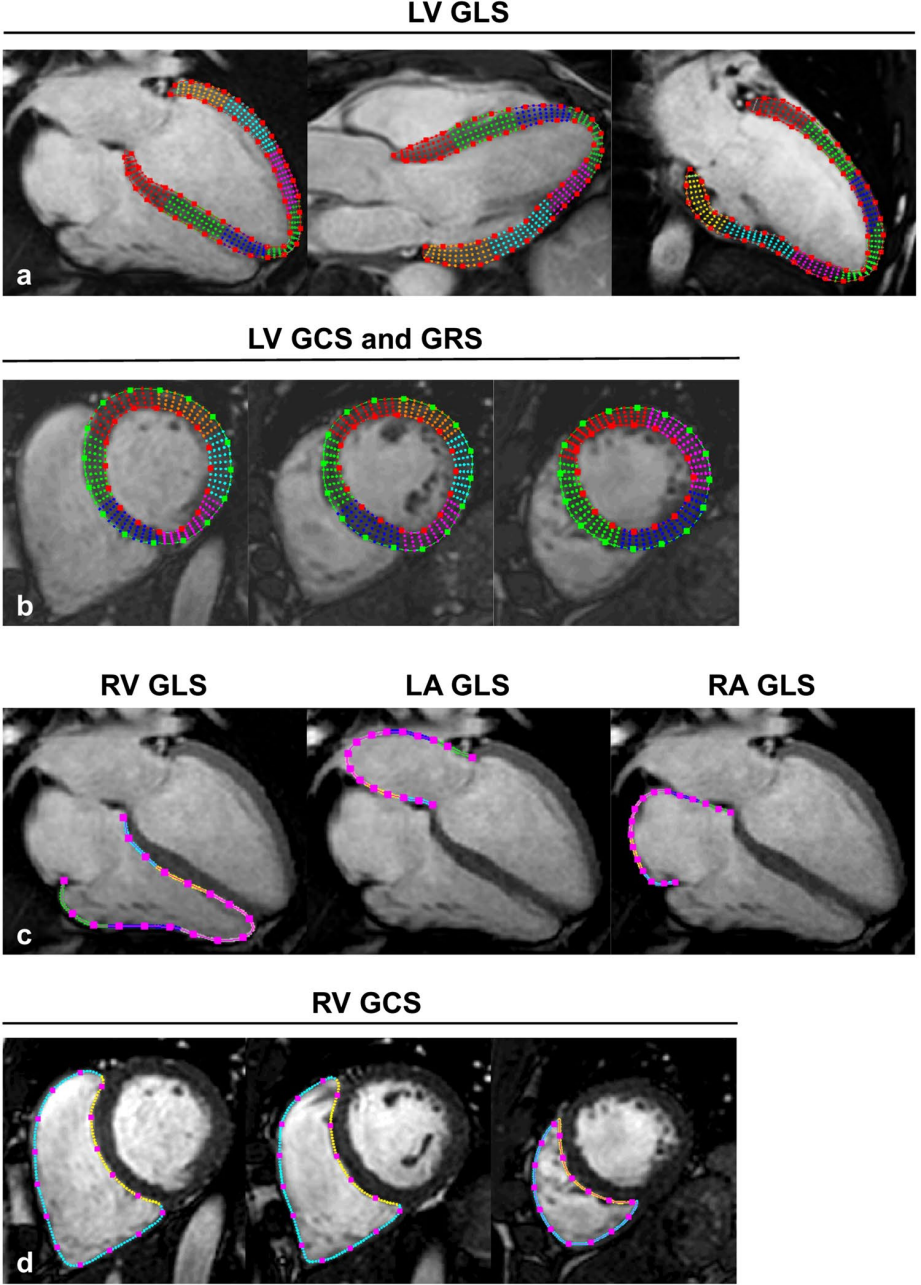
Depiction of LV endo- and epicardial, and RV, LA, and RA endocardial contours to determine LV GLS (**a**); LV GCS and GRS (**b**); RV, LA, and RA GLS (**c**); as well as RV GCS (**d**). Strain values were generated by feature-tracking CMR using the Segment software, which automatically propagates the manually drawn contours through all cardiac phases of the long- and short-axis cardiac cine series

Myocardial strain was analyzed on cine images using feature-tracking software (Segment, version 2.1.R.6108, Medviso) as previously reported [15]. In short, this software analyzes myocardial strain by computing interframe deformation fields using an endocardial tracking strategy based on non-rigid image registration. LV global longitudinal strain (GLS) was measured on 3 long-axis cine series using 2-, 3-, and 4-chamber views (Fig. 1a), whereas LV global circumferential strain (GCS) and radial strain (GRS) were measured on three short-axis cine series (apical, midventricular, and basal) (Fig. 1b) [17]. Long-axis 4-chamber and three short-axis series were used to calculate RV GLS and GCS (Fig. 1c, d); long-axis 4-chamber series were applied for RV free wall (FW) GLS [17]. LA and RA endocardial contours were manually delineated on end-diastolic images and propagated throughout the cardiac cycle generating longitudinal strain (Fig. 1c) [18].

### Statistical analysis

Statistical analysis was performed with commercially available software (GraphPad Prism, version 9.00) and SPSS for Windows (version 21.0, IBM SPSS Inc.). All CMR data are given as the mean of two observers (H.C. and M.W.). Continuous variables were checked for normality using the D’Agostino-Pearson omnibus normality test. Normally distributed data are presented as mean ± SD and categorical data are presented as absolute numbers and percentage. Normally distributed data were compared using the unpaired or paired Student’s *t*-test. If normal distribution was not given, the Mann–Whitney or the Wilcoxon matched-pairs signed rank tests were used. Categorical variables were compared using the *χ*^2^ test or Fischer’s exact test as appropriate. Statistical significance was defined as *p* < 0.05.

## Results

### Baseline characteristics of triathletes compared to controls

There were no differences in age, sex distribution, body surface area, blood biomarkers, and blood pressure (BP) between controls and triathletes (Table 1). Triathletes had a lower heart rate (*p* < 0.0001), but LV cardiac index (*p* = 0.927), LVEF (*p* = 0.766), and RVEF (*p* = 0.606) were similar. LV mass (*p* < 0.0001), ventricular, and atrial volumes were higher in triathletes (Table 1). LV GLS (*p* < 0.05), GCS (*p* < 0.01), and GRS (*p* < 0.05) as well as RV GLS (*p* < 0.0001) and GCS (*p* < 0.0001), RV FW GLS (*p* < 0.05), and RA GLS (*p* < 0.0001) were lower in triathletes (Table 1). LA GLS (*p* = 0.288) was similar between triathletes and controls. Triathletes had a lower native T1 value (*p* < 0.0001), but there were no differences in T2 and ECV values. Baseline CMR revealed LGE indicative of focal myocardial fibrosis in 11 of 40 (28%) male triathletes, but in none of the male controls (*p* < 0.01). None of the female triathletes had LGE.

**Table 1:** Demographics and CMR parameters of all triathletes at baseline compared to sedentary controls and post-race alterations in triathletes

	Controls (*n* = 28)	Baseline All triathletes (*n* = 50)	Post-race All triathletes (*n* = 50)	*p* value
Clinical parameters				
Age, years	42 ± 11	45 ± 10	-	-
Female, %	6 (21)	10 (20)	-	-
Body surface area, m^2^	1.94 ± 0.19	1.94 ± 0.19	1.93 ± 0.19	< 0.01
Troponin T, pg/ml	5 ± 3	6 ± 4	51 ± 77	< 0.0001
NT-proBNP, pg/ml	41 ± 24	45 ± 73	116 ± 99	< 0.0001
CK, U/l	182 ± 179	188 ± 145	478 ± 316	< 0.0001
CK-MB, U/l	4 ± 8	10 ± 14	32 ± 19	< 0.0001
Systolic BP at rest, mmHg	121 ± 15	126 ± 15	-	-
Diastolic BP at rest, mmHg	80 ± 18	84 ± 9	-	-
CMR—left heart				
Heart rate, bpm	66 ± 11	54 ± 8^‡^	68 ± 10	< 0.0001
LV cardiac index, l/min/m^2^	3.20 ± 0.82	3.30 ± 0.66	3.91 ± 0.60	< 0.0001
LVEF, %	63 ± 8	62 ± 6	63 ± 7	0.607
LV mass index, g/m^2^	65 ± 11	78 ± 11^‡^	78 ± 12	0.499
LVEDVi, ml/m^2^	79 ± 13	98 ± 17^‡^	93 ± 15	< 0.001
LVESVi, ml/m^2^	30 ± 10	37 ± 9^†^	35 ± 11	0.084
LVSVi, ml/m^2^	49 ± 8	61 ± 10^‡^	58 ± 10	0.100
LAEDVi, ml/m^2^	13 ± 4	20 ± 8^‡^	16 ± 6	< 0.01
LAESVi, ml/m^2^	33 ± 7	47 ± 12^‡^	37 ± 10	< 0.0001
CMR—right heart				
RVEF, %	60 ± 7	59 ± 9	60 ± 8	0.552
RVEDVi, ml/m^2^	80 ± 14	101 ± 19^‡^	97 ± 18	< 0.05
RVESVi, ml/m^2^	32 ± 10	41 ± 14^†^	40 ± 14	0.065
RVSVi, ml/m^2^	48 ± 7	60 ± 10^‡^	57 ± 9	0.296
RAEDVi, ml/m^2^	19 ± 5	28 ± 10^‡^	28 ± 10	0.460
RAESVi, ml/ m^2^	36 ± 7	52 ± 14^‡^	47 ± 12	< 0.01
CMR—strain				
LV GLS, %	− 19 ± 2	− 18 ± 2*	− 17 ± 2	0.054
LV GCS, %	− 19 ± 4	− 16 ± 4^†^	− 18 ± 4	< 0.05
LV GRS, %	45 ± 11	39 ± 11*	44 ± 11	< 0.01
RV GLS, %	− 22 ± 3	− 19 ± 3^‡^	− 19 ± 3	0.668
RV GCS, %	− 10 ± 3	− 7 ± 2^‡^	− 8 ± 3	< 0.001
RV FW GLS, %	− 24 ± 7	− 21 ± 6*	− 21 ± 8	0.309
LA GLS, %	28 ± 5	30 ± 8	24 ± 6	< 0.0001
RA GLS, %	33 ± 7	25 ± 6^‡^	24 ± 6	0.519
CMR—mapping				
Native T1, ms	1034 ± 32	987 ± 27^‡^	990 ± 22	0.486
Native T2, ms	53 ± 3	53 ± 2	53 ± 3	0.458
ECV, %	25.6 ± 4.1	25.7 ± 2.0	-	-

Numbers are mean ± SD for continuous and *n* (%) for categorical data

^*^p < 0.05; ^†^*p* < 0.01; or ^‡^*p* < 0.0001 for all triathletes vs. controls

Baseline and post-race data were partly reported in previous publications as indicated in the “Methods and materials” section

Abbreviations: *BP*, blood pressure; *EF*, ejection fraction; *GCS*, global circumferential strain; *GLS*, global longitudinal strain; *GRS*, global radial strain; *LA*, left atrial; *LAEDVi*, left atrial end-diastolic volume index; *LAESVi*, left atrial end-systolic volume index; *LV*, left ventricular; *LVEDVi*, left ventricular end-diastolic volume index; *LVESVi*, left ventricular end-systolic volume index; *RA*, right atrial; *RAEDVi*, right atrial end-diastolic volume index; *RAESVi*, right atrial end-systolic volume index; *RV*, right ventricular; *RVEDVi*, right ventricular end-diastolic volume index; *RVESVi*, right ventricular end-systolic volume index; *RV FW*, right ventricular free wall

### Post-race changes in all triathletes

**Fig. 2:**
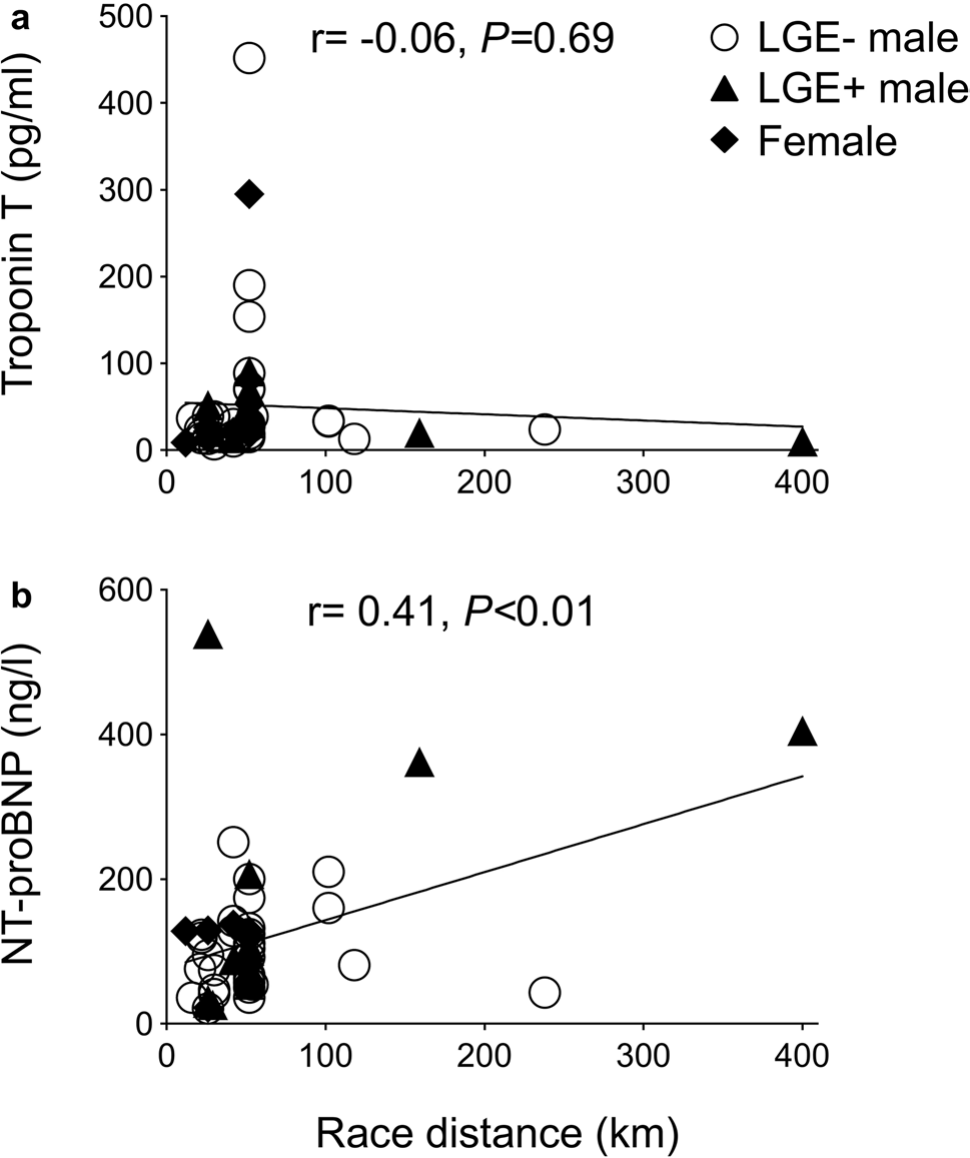
Correlation between troponin T (**a**) and NT-proBNP (**b**) and the race cumulative distance

Cardiac biomarkers increased in all triathletes including troponin T, NT-proBNP, and creatine kinase MB (Table 1). There was no correlation between troponin T and the race distance (*r* =  − 0.06, *p* = 0.69; Fig. 2a), but NT-proBNP showed a positive correlation (*r* = 0.41, *p* < 0.01; Fig. 2b). CMR revealed post-race volume decrease in all cardiac chambers, whereas heart rate and cardiac index increased (Table 1). LV (*p* = 0.607) and RV ejection fractions (*p* = 0.552) remained constant.

**Fig. 3:**
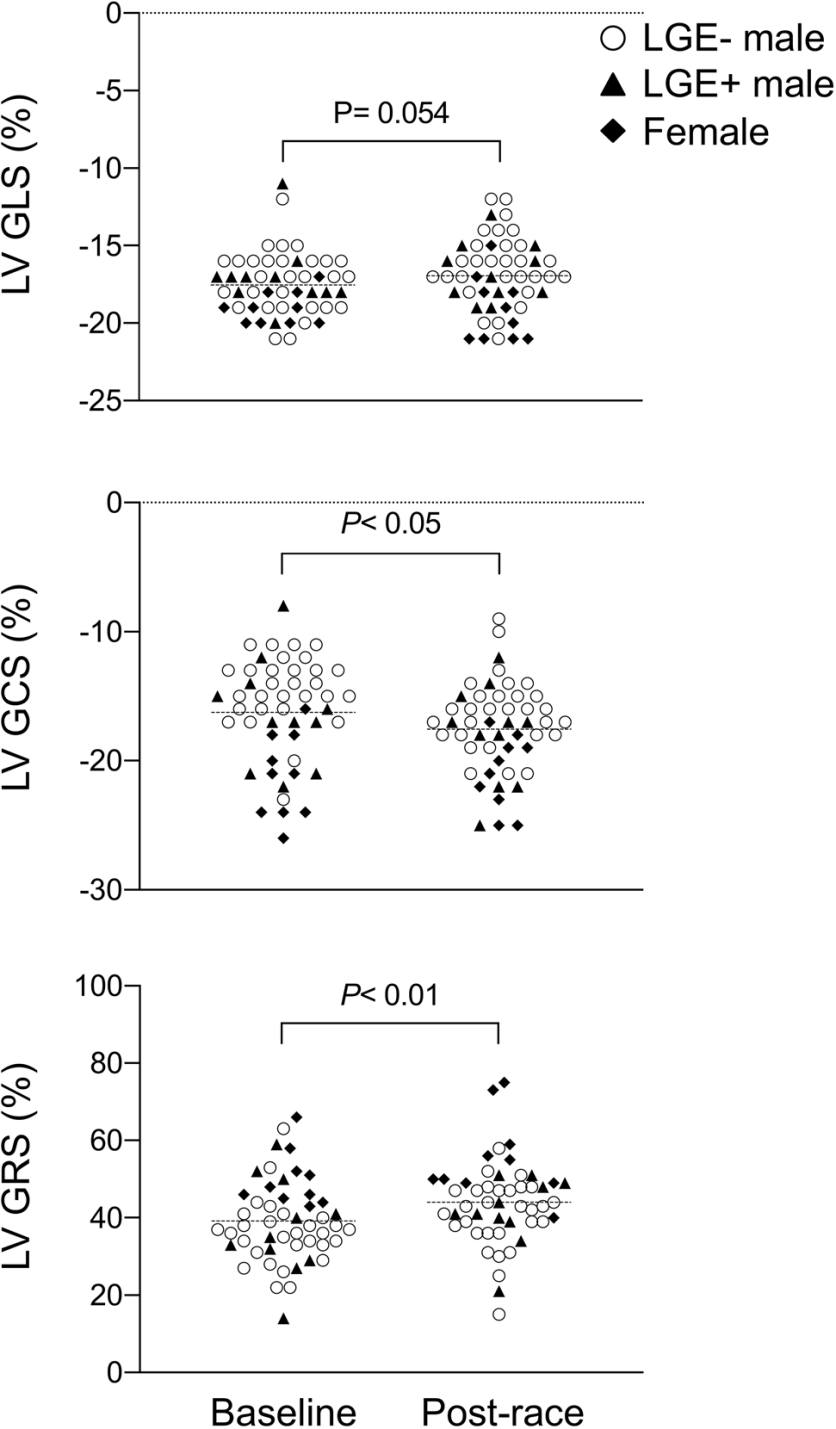
Changes of LV GLS, GCS, and GRS following an acute bout of endurance exercise in male and female triathletes as assessed by feature-tracking CMR

**Fig. 4:**
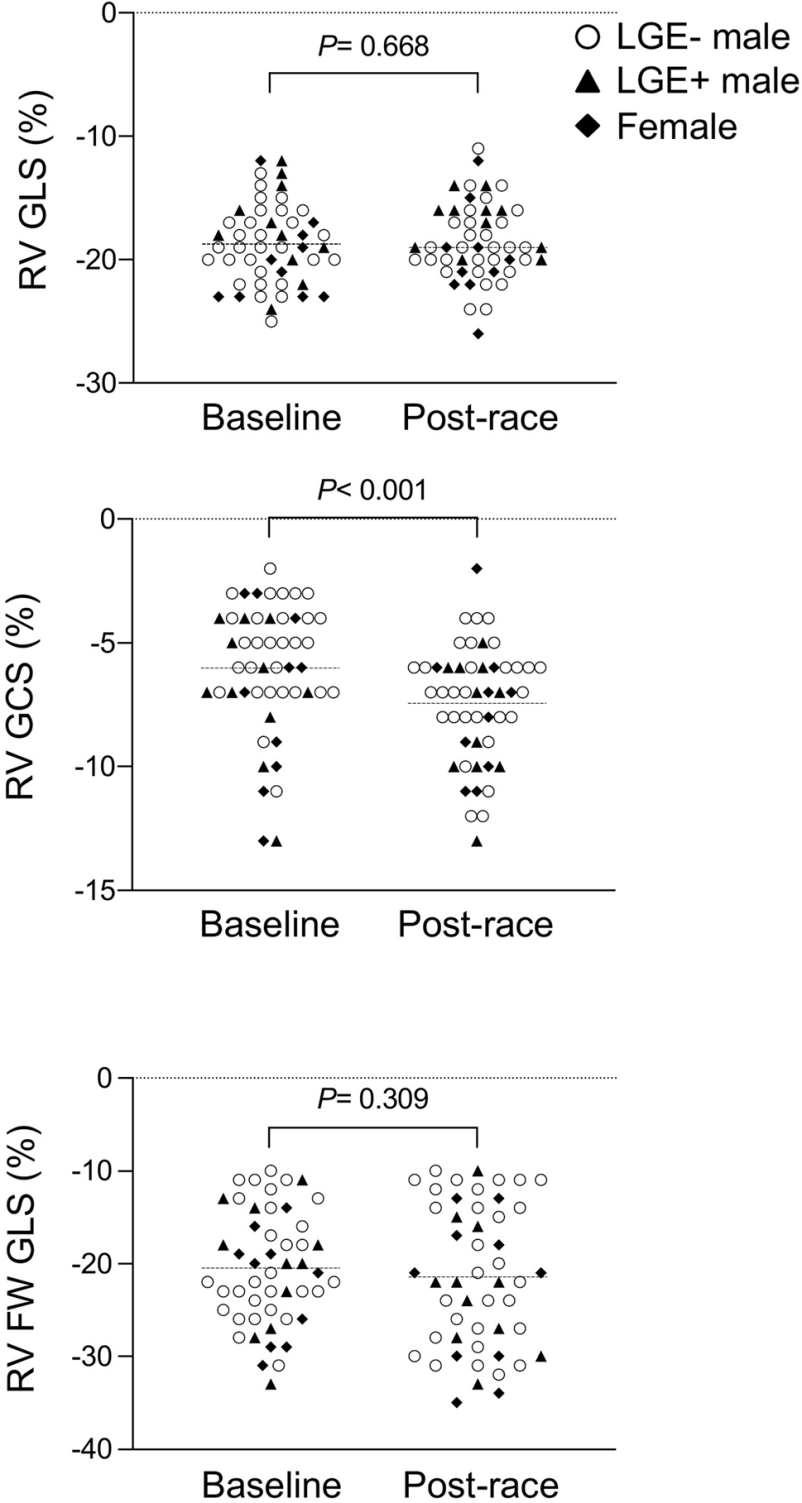
Changes of RV GLS and GCS and RV free wall GLS following an acute bout of endurance exercise in male and female triathletes as assessed by feature-tracking CMR

**Fig. 5:**
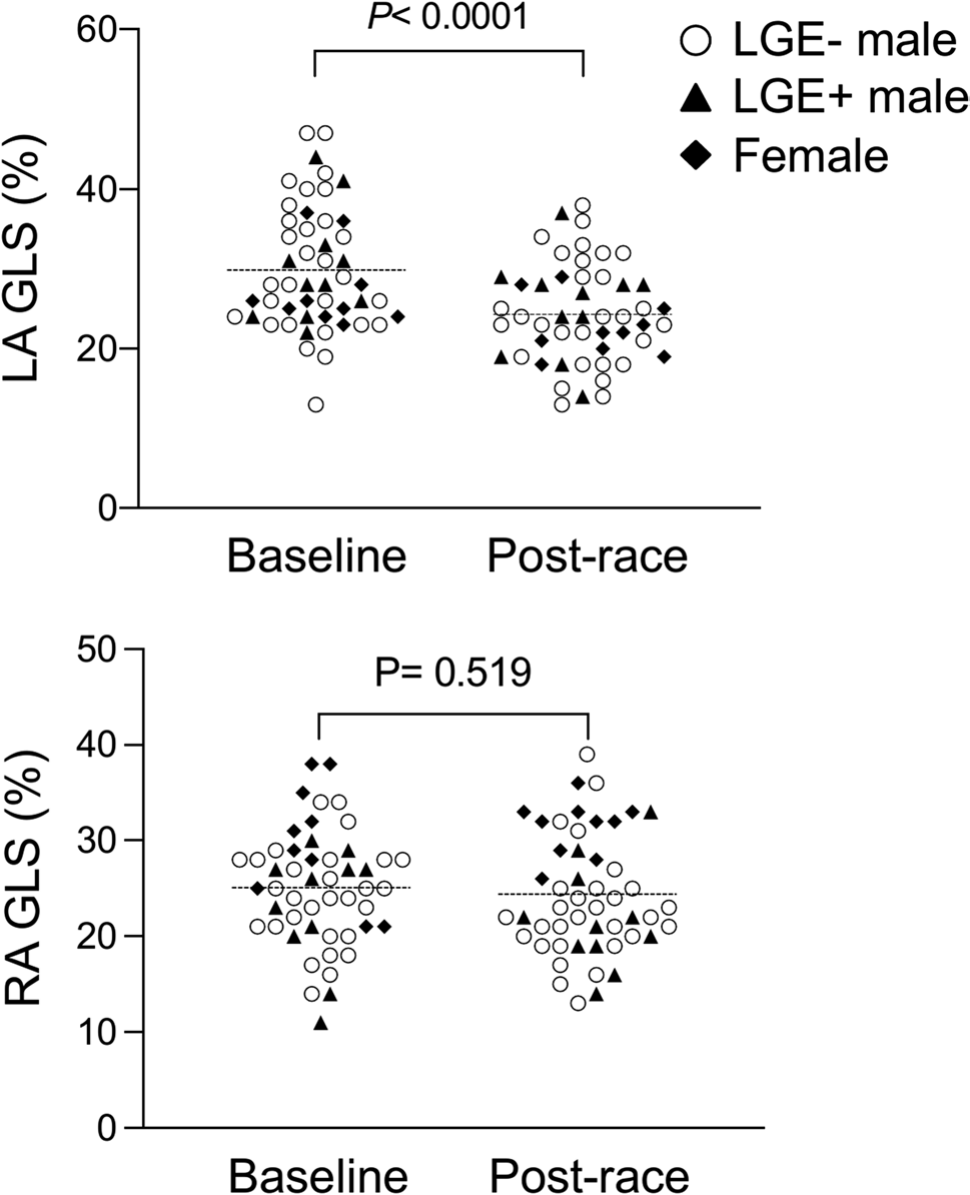
Changes of LA and RA GLS following an acute bout of endurance exercise in male and female triathletes as assessed by feature-tracking CMR

For clarity, systolic GLS and GCS values are negative by convention; thus, fewer negative values indicate decreased contractility. Systolic GRS on the other hand has positive values. In this study, post-race LV GLS decreased by tendency (− 18 ± 2 vs. − 17 ± 2%, *p* = 0.054), whereas GCS (− 16 ± 4 vs. − 18 ± 4%, *p* < 0.05) and GRS increased (39 ± 11 vs. 44 ± 11%, *p* < 0.01; Table 1, Fig. 3). Post-race RV GLS (*p* = 0.668) and FW GLS (*p* = 0.309) remained constant, RV GCS increased (− 7 ± 2 vs. − 8 ± 3%, *p* < 0.001; Table 1, Fig. 4), and LA GLS decreased (30 ± 8 vs. 24 ± 6%, *p* < 0.0001; Table 1, Fig. 5). Post-race RA GLS (*p* = 0.519), native T1 (*p* = 0.486), and T2 (*p* = 0.458) were stable.

### Post-race correlations between blood biomarkers and myocardial strain

Post-race troponin T correlated with LV GLS (*r* = 0.30, *p* < 0.05), LV GCS (*r* = 0.37, *p* < 0.01), and LV GRS (*r* =  − 0.39, *p* < 0.01), indicating decrease of myocardial contractility with increasing post-race troponin T. There were no correlations with LA GLS (*r* =  − 0.20, *p* = 0.155), RA GLS (*r* = 0.10, *p* = 0.506), T1 (*r* =  − 0.11, *p* = 0.451), and T2 (*r* =  − 0.26, *p* = 0.069). Post-race NT-proBNP did not correlate with any of the strain parameters.

### Post-race changes of myocardial strain in male triathletes

Post-race LV (*p* = 0.476) and RV EF (*p* = 0.499) were unchanged. Post-race LV GLS decreased (− 17 ± 2 vs. − 16 ± 2%, *p* < 0.05), whereas GCS (− 15 ± 3 vs. − 17 ± 3%, *p* < 0.01) and GRS increased (36 ± 10 vs. 41 ± 9%, *p* < 0.05; Table 2). Post-race RV GLS (*p* = 0.724) and FW GLS (*p* = 0.331) remained constant and GCS increased (− 6 ± 3 vs. − 7 ± 2%, *p* < 0.001). LA GLS (28 ± 7 vs. 23 ± 5%, *p* < 0.001) decreased. Post-race RA GLS remained unchanged (*p* = 0.266; Table 2).

**Table 2:** Demographics and CMR parameters of male triathletes at baseline compared to sedentary controls and post-race alterations in triathletes

	Controls (*n* = 22)	Baseline Men (*n* = 40)	Post-race Men (*n* = 40)	*p* value
Clinical parameters				
Age, years	40 ± 12	44 ± 9	-	**-**
Body surface area, m^2^	2.00 ± 0.14	2.01 ± 0.14	2.00 ± 0.14	< 0.01
Troponin T, pg/ml	5 ± 3	6 ± 4	50 ± 75	< 0.001
NT-proBNP, pg/ml	39 ± 25	45 ± 79	121 ± 108	< 0.0001
CK, U/l	210 ± 191	206 ± 156	503 ± 289	< 0.0001
CK-MB, U/l	5 ± 8	11 ± 15	33 ± 18	< 0.0001
Systolic BP at rest, mmHg	121 ± 15	126 ± 12	-	-
Diastolic BP at rest, mmHg	84 ± 11	84 ± 9	-	-
Age, years	40 ± 12	44 ± 9	-	**-**
CMR—left heart				
Heart rate, bpm	66 ± 12	54 ± 8^ll^	68 ± 10	< 0.0001
LV cardiac index, l/min/m^2^	3.31 ± 0.89	3.29 ± 0.64	3.93 ± 0.58	< 0.0001
LVEF, %	62 ± 9	61 ± 6	62 ± 7	0.476
LV mass index, g/m^2^	68 ± 8	80 ± 10^ll^	81 ± 11	0.486
LVEDVi, ml/m^2^	82 ± 12	102 ± 16^ll^	96 ± 15	< 0.001
LVESVi, ml/m^2^	32 ± 10	39 ± 9^†^	37 ± 10	0.066
LVSVi, ml/m^2^	50 ± 8	62 ± 11^ll^	59 ± 10	0.113
LAEDVi, ml/m^2^	13 ± 4	20 ± 9^‡^	17 ± 6	< 0.01
LAESVi, ml/m^2^	32 ± 8	48 ± 13^ll^	37 ± 10	< 0.0001
CMR—right heart				
RVEF, %	59 ± 7	58 ± 9	59 ± 8	0.499
RVEDVi, ml/m^2^	84 ± 13	105 ± 19^ll^	100 ± 19	0.051
RVESVi, ml/m^2^	34 ± 10	45 ± 14^†^	42 ± 14	< 0.05
RVSVi, ml/m^2^	49 ± 6	60 ± 11^‡^	58 ± 10	0.300
RAEDVi, ml/m^2^	20 ± 5	30 ± 10^†^	29 ± 11	0.633
RAESVi, ml/ m^2^	38 ± 7	52 ± 14^†^	47 ± 12	< 0.01
CMR—strain				
LV GLS, %	− 18 ± 2	− 17 ± 2	− 16 ± 2	< 0.05
LV GCS, %	− 18 ± 4	− 15 ± 3^†^	− 17 ± 3	< 0.01
LV GRS, %	44 ± 11	36 ± 10^†^	41 ± 9	< 0.05
RV GLS, %	− 22 ± 3	− 18 ± 3^‡^	− 18 ± 3	0.724
RV GCS, %	− 10 ± 3	− 6 ± 3^ll^	− 7 ± 2	< 0.001
RV FW GLS, %	− 23 ± 7	− 20 ± 6	− 21 ± 8	0.331
LA GLS, %	28 ± 5	28 ± 7	23 ± 5	< 0.001
RA GLS, %	32 ± 7	24 ± 5^ll^	23 ± 6	0.266
CMR—mapping				
Native T1, ms	1024 ± 30	983 ± 28^ll^	987 ± 20	0.512
Native T2, ms	52 ± 4	53 ± 3	53 ± 3	0.854
ECV, %	24.9 ± 3	24.9 ± 1.5	-	-

Numbers are mean ± SD for continuous and *n* (%) for categorical data

^*^p < 0.05; ^†^*p* < 0.01; ^‡^*p* < 0.001; or ^ll^*p* < 0.0001 for male triathletes vs. controls

Baseline and post-race data were partly reported in previous publications as indicated in the “Methods and materials” section

Abbreviations: *BP*, blood pressure; *EF*, ejection fraction; *GCS*, global circumferential strain; *GLS*, global longitudinal strain; *GRS*, global radial strain; *LA*, left atrial; *LAEDVi*, left atrial end-diastolic volume index; *LAESVi*, left atrial end-systolic volume index; *LV*, left ventricular; *LVEDVi*, left ventricular end-diastolic volume index; *LVESVi*, left ventricular end-systolic volume index; *RA*, right atrial; *RAEDVi*, right atrial end-diastolic volume index; *RAESVi*, right atrial end-systolic volume index; *RV*, right ventricular; *RVEDVi*, right ventricular end-diastolic volume index; *RVESVi*, right ventricular end-systolic volume index; *RV FW*, right ventricular free wall

### Post-race changes of myocardial strain in female triathletes

Post-race LV (*p* = 0.864) and RV EF (*p* = 0.909) were constant. Post-race LV GLS (*p* = 0.891), GCS (*p* = 0.861), and GRS (*p* = 0.147) as well as RV GLS (*p* = 0.790), GCS (*p* = 0.651), FW GLS (*p* = 0.744), and RA GLS (*p* = 0.505) remained unchanged (Table 3). Post-race LA GLS (37 ± 7 vs. 31 ± 5%, *p* < 0.05) decreased (Table 3).

**Table 3:** Demographics and CMR parameters of female triathletes at baseline compared to sedentary controls and post-race alterations in triathletes

	Controls (*n* = 6)	Baseline Women (*n* = 10)	Post-race Women (*n* = 10)	*p* value
Clinical parameters				
Age, years	47 ± 6	46 ± 11	-	**-**
Body surface area, m^2^	1.71 ± 0.17	1.68 ± 0.11	1.66 ± 0.08	0.273
Troponin T, pg/ml	3 ± 1	4 ± 2	56 ± 87	0.089
NT-proBNP, pg/ml	51 ± 7	45 ± 40	97 ± 41	< 0.01
CK, U/l	69 ± 20	117 ± 50	376 ± 411	0.085
CK-MB, U/l	3 ± 2	3 ± 7	27 ± 21	< 0.01
Systolic BP at rest, mmHg	123 ± 17	128 ± 23	-	-
Diastolic BP at rest, mmHg	84 ± 12	85 ± 9	-	-
Age, years	47 ± 6	46 ± 11	-	**-**
CMR—left heart				
LVEF, %	67 ± 6	67 ± 4	67 ± 8	0.864
Heart rate, bpm	65 ± 8	56 ± 8*	70 ± 9	< 0.01
LV cardiac index, l/min/m^2^	2.89 ± 0.51	3.16 ± 0.80	3.80 ± 0.71	0.097
LV mass index, g/m^2^	53 ± 8	67 ± 9^†^	67 ± 9	0.909
LVEDVi, ml/m^2^	68 ± 8	83 ± 9^†^	81 ± 9	0.340
LVESVi, ml/m^2^	22 ± 5	27 ± 4*	27 ± 7	0.928
LVSVi, ml/m^2^	45 ± 8	56 ± 8*	54 ± 9	0.647
LAEDVi, ml/m^2^	11 ± 1	18 ± 6*	16 ± 5	0.205
LAESVi, ml/m^2^	35 ± 5	42 ± 10	39 ± 8	0.212
CMR—right heart				
RVEF, %	63 ± 4	65 ± 7	66 ± 7	0.909
RVEDVi, ml/m^2^	68 ± 7	87 ± 12^†^	85 ± 9	0.523
RVESVi, ml/m^2^	25 ± 4	30 ± 7	30 ± 9	0.796
RVSVi, ml/m^2^	43 ± 6	57 ± 11*	55 ± 5	0.784
RAEDVi, ml/m^2^	16 ± 3	25 ± 7*	23 ± 7	0.415
RAESVi, ml/ m^2^	31 ± 4	50 ± 11	47 ± 9	0.225
CMR—strain				
LV GLS, %	− 21 ± 1	− 19 ± 1^†^	− 19 ± 2	0.891
LV GCS, %	− 23 ± 2	− 21 ± 3	− 21 ± 3	0.861
LV GRS, %	52 ± 9	50 ± 7	56 ± 11	0.147
RV GLS, %	− 23 ± 2	− 20 ± 4	− 20 ± 4	0.790
RV GCS, %	− 10 ± 1	− 7 ± 3	− 8 ± 3	0.651
RV FW GLS, %	− 27 ± 7	− 22 ± 6	− 23 ± 8	0.744
LA GLS, %	30 ± 5	37 ± 7*	31 ± 5	< 0.05
RA GLS, %	38 ± 7	30 ± 6*	31 ± 3	0.505
LV GLS, %	− 21 ± 1	− 19 ± 1^†^	− 19 ± 2	0.891
CMR—mapping				
Native T1, ms	1065 ± 15	999 ± 20^‡^	1001 ± 25	0.823
Native T2, ms	55 ± 1	53 ± 2	52 ± 2	0.218
ECV, %	30.2 ± 3.9	27.4 ± 1.5	-	-

Numbers are mean ± SD for continuous and *n* (%) for categorical data

^*^p < 0.05; ^†^*p* < 0.01; or ^‡^*p* < 0.0001 for female triathletes vs. controls

Baseline and post-race data were partly reported in previous publications as indicated in the “Methods and materials” section

Abbreviations: *BP*, blood pressure; *EF*, ejection fraction; *GCS*, global circumferential strain; *GLS*, global longitudinal strain; *GRS*, global radial strain; *LA*, left atrial; *LAEDVi*, left atrial end-diastolic volume index; *LAESVi*, left atrial end-systolic volume index; *LV*, left ventricular; *LVEDVi*, left ventricular end-diastolic volume index; *LVESVi*, left ventricular end-systolic volume index; *RA*, right atrial; *RAEDVi*, right atrial end-diastolic volume index; *RAESVi*, right atrial end-systolic volume index; *RV*, right ventricular; *RVEDVi*, right ventricular end-diastolic volume index; *RVESVi*, right ventricular end-systolic volume index; *RV FW*, right ventricular free wall

### *Post-race changes of myocardial strain in LGE* + *male triathletes*

Baseline CMR revealed non-ischemic myocardial fibrosis in 11 (28%) male triathletes. The LGE pattern was previously reported in detail [14, 15]. Briefly, predominantly anterolateral, inferolateral, and inferior segments of the basal LV wall were LGE + , a distribution typical for myocarditis.

Post-race LV (*p* = 0.999) and RV ejection fractions (*p* = 0.999) were unchanged in LGE + male triathletes. Post-race LV GLS (− 17 ± 2 vs. − 17 ± 2%, *p* = 0.669), GCS (− 16 ± 4 vs. − 18 ± 4%, *p* = 0.219), and GRS (38 ± 13 vs. 42 ± 9%, *p* = 0.267) as well as RV GLS (− 20 ± 4 vs. − 18 ± 7%, *p* = 0.227), GCS (− 8 ± 3 vs. − 7 ± 4%, *p* = 0.616), FW GLS (− 20 ± 7 vs. − 23 ± 7%, *p* = 0.356), and RA GLS (23 ± 6 vs. 22 ± 5%, *p* = 0.585) all remained unchanged (Table 4). LA GLS (25 ± 6 vs. 23 ± 4%, *p* = 0.276) was constant after the endurance race (Table 4). Strain values of LGE − male triathletes are detailed in Supplemental Table 1.

**Table 4:** Demographics and CMR parameters of LGE + triathletes at baseline and post-race

	Baseline LGE + (*n* = 11)	Post-race LGE + (*n* = 11)	*p* value
Clinical parameters			
Body surface area, m^2^	1.97 ± 0.16	1.95 ± 0.15	< 0.05
Troponin T, pg/ml	7 ± 6	39 ± 25	< 0.01
NT-proBNP, pg/ml	83 ± 144	174 ± 179	< 0.05
CK, U/l	126 ± 48	388 ± 210	< 0.01
CK-MB, U/l	4 ± 8	30 ± 21	< 0.01
Systolic BP at rest, mmHg	128 ± 16	-	-
Diastolic BP at rest, mmHg	83 ± 9	-	-
CMR—left heart			
Heart rate, bpm	55 ± 10	65 ± 9	< 0.001
LV cardiac index, l/min/m^2^	3.39 ± 0.76	3.92 ± 0.52	< 0.05
LVEF, %	63 ± 8	63 ± 7	0.999
LV mass index, g/m^2^	87 ± 7	87 ± 8	0.638
LVEDVi, ml/m^2^	101 ± 15	97 ± 17	0.187
LVESVi, ml/m^2^	37 ± 9	37 ± 11	0.999
LVSVi, ml/m^2^	62 ± 12	61 ± 12	0.710
LAEDVi, ml/m^2^	23 ± 8	21 ± 5	0.324
LAESVi, ml/m^2^	53 ± 14	44 ± 10	< 0.05
CMR—right heart			
RVEF, %	61 ± 10	61 ± 8	0.999
RVEDVi, ml/m^2^	106 ± 21	98 ± 20	0.083
RVESVi, ml/m^2^	42 ± 14	38 ± 12	0.327
RVSVi, ml/m^2^	65 ± 14	60 ± 11	0.194
RAEDVi, ml/m^2^	32 ± 13	31 ± 11	0.393
RAESVi, ml/ m^2^	54 ± 17	52 ± 15	0.377
CMR—strain			
LV GLS, %	− 17 ± 2	− 17 ± 2	0.669
LV GCS, %	− 16 ± 4	− 18 ± 4	0.219
LV GRS, %	38 ± 13	42 ± 9	0.267
RV GLS, %	− 20 ± 4	− 18 ± 7	0.227
RV GCS, %	− 8 ± 3	− 7 ± 4	0.616
RV FW GLS, %	− 20 ± 7	− 23 ± 7	0.356
LA GLS, %	25 ± 6	23 ± 4	0.276
RA GLS, %	23 ± 6	22 ± 5	0.585
CMR—mapping			
Native T1, ms	997 ± 37	993 ± 22	0.585
Native T2, ms	53 ± 3	53 ± 2	0.815
ECV, %	26.3 ± 2.2	-	-

Numbers are mean ± SD for continuous and *n* (%) for categorical data

Baseline and post-race data were partly reported in previous publications as indicated in the “Methods and materials” section

Abbreviations: *BP*, blood pressure; *EF*, ejection fraction; *GCS*, global circumferential strain; *GLS*, global longitudinal strain; *GRS*, global radial strain; *LA*, left atrial; *LAEDVi*, left atrial end-diastolic volume index; *LAESVi*, left atrial entad-systolic volume index; *LV*, left ventricular; *LVEDVi*, left ventricular end-diastolic volume index; *LVESVi*, left ventricular end-systolic volume index; *RA*, right atrial; *RAEDVi*, right atrial end-diastolic volume index; *RAESVi*, right atrial end-systolic volume index; *RV*, right ventricular; *RVEDVi*, right ventricular end-diastolic volume index; *RVESVi*, right ventricular end-systolic volume index; *RV FW*, right ventricular free wall

## Discussion

This prospective study analyzed biventricular and biatrial myocardial strain changes in male and female triathletes following an acute bout of endurance exercise. Further, strain analysis in a subgroup of male triathletes with focal myocardial fibrosis (LGE +) was performed. The major findings are as follows: (1) Left ventricular GLS was slightly reduced, whereas GCS and GRS increased following an endurance race; (2) Right ventricular GLS and free wall longitudinal strain remained constant, whereas GCS increased post-race; (3) Left atrial longitudinal strain was decreased post-race, and right atrial longitudinal strain remained constant; and (4) Male triathletes with focal myocardial fibrosis (LGE +) had constant baseline and post-race biventricular and biatrial strain parameters.

### Alterations in left ventricular strain after an endurance race

Stewart et al observed in a recent study that LV GLS decreased in 10 recreationally active men after a 90-min high-intensity cycling exercise, but not after a 120-min moderate-intensity cycling [8]. Vitiello et al reported that LV GLS and GCS decreased in a cohort of 16 young men following a 180-min strenuous cycling exercise, but not GRS [19]. Further, Aengevaeren et al used CMR to investigate the influence of a marathon run on myocardial injury and cardiomyocyte integrity in highly trained males, and reported that LV GCS was attenuated post-marathon and did not recover within 2 weeks [20]. In concordance with another recent study by Cavigli et al [21], we also observed a post-race tendency of LV GLS reduction in triathletes and constant LVEF. Conversely, LV GCS and GRS were increased. There might be several explanations for this discrepancy. First, the acquisition time point of post-race CMR in our study was 2.3 h (range 1–5) and thus later than in the aforementioned echocardiographic studies, which might have provided a longer recovery period. Second, the duration and components of exercise were different. Stewart et al and Vitiello et al used a cycle ergometer in a strictly controlled in-door environment [8, 19]. Further, a marathon is an utterly strenuous and extended duration continuous exercise, while our triathletes participated in open-air races with varied distances and presumably less cardiac load. Third, strain parameters are not only a measure of intrinsic myocardial contractility, but are also influenced by cardiac load (i.e., end-systolic and diastolic blood pressure, LV volume) and structure [22]. It is worth noting that a recent study aimed to compare the accuracy and reproducibility of FT-CMR and speckle tracking echocardiography (STE).

in endurance athletes [23]. The authors concluded that biventricular longitudinal strain values were lower when assessed by FT-CMR compared to STE and both methods were statistically comparable and concordant when measuring LV strain, but not RV strain [23]. Thus, while comparing strain studies, the potential influence of different imaging techniques needs to be considered. The increase in LV GCS and GRS in the current study might be a compensatory mechanism for the loss of longitudinal mechanical function, as observed in early stages of progressive myocardial disease [4].

### Alterations in right ventricular strain after an endurance race

A transient deterioration of RVEF and RV GLS in athletes after an endurance race has been previously demonstrated [3, 24]. However, a more recent study by Cavigli et al investigated the acute impact of an ultra-marathon on the RV and could not identify any alterations in RV GLS [21]. Similar to Cavigli et al, we observed that RV GLS and RV FW GLS did not change between baseline and post-race [21]. Our study adds to the current scientific knowledge demonstrating that RV GCS increases after an endurance race, again as a possible intrinsic compensatory mechanism [4]. The RV end-diastolic volume decreased post-race, possibly followed by an adaptation of myocardial deformation to maintain normal RVEF according to the Frank-Starling mechanism [25]. There may also be alterations in RV strain during different phases of post-race recovery, and the recovery time may depend on the duration and intensity of the endurance exercise.

### Reduced left atrial and constant right atrial strain after an endurance race

The left atrium (LA) plays a crucial role in regulating LV filling by functioning as a reservoir for pulmonary venous return during ventricular systole and a booster pump for strengthening ventricular filling during late diastole [26]. D’Ascenzi et al reported in an echocardiographic study that while the LA volume in elite soccer players is increased compared to controls, LA GLS was similar [27]. The current study confirms these findings and extends the knowledge on LA adaptation following an endurance race demonstrating a volume decrease and attenuation in GLS. Our findings are consistent with previous echocardiographic data showing a decrease in LA volume and a reduction in peak atrial longitudinal strain post-exercise [28]. Consistently, Oxborough et al found that the indices of LA deformation and volume were reduced immediately post-race [29]. Even though immediate post-exercise echocardiography data is mostly reported, cardiac recovery might actually exceed 24 h [30]. The acquisition time point of post-race CMR in the current study was within 5 h, and exercise-induced cardiac alterations might have not fully subsided.

Further, we observed lower baseline RA strain in triathletes compared to controls. This finding is consistent with a prior echocardiographic study by D’Ascenzi et al, which implies that the reduction of RA GLS in athletes represents a physiological adaptation rather than an abnormality [31]. Athlete’s atria are at lower strain and larger volumes facilitating a larger atrial stroke volume reserve [32]. Post-race RA GLS did not change in this study, which is partially in line with the study by Sanz-de et al, who investigated the exercise-dose-dependent impairment in atrial function [33]. An exercise load of 14 km showed no changes of post-race RA strain, but higher exercise loads induced impairment in atrial contractile function [33].

### Ventricular and atrial strain in LGE + male triathletes at baseline and after an endurance race

It has been suggested that repetitive and sustained exposure to high-intensity exercise might induce cardiac microdamage associated with development of myocardial fibrosis [34]. A notion that is supported by the high prevalence of focal myocardial fibrosis in athletes detected by CMR LGE imaging [3, 35]. A subgroup analysis of male triathletes with focal myocardial fibrosis (LGE +) showed no differences at baseline and post-race regarding biventricular and biatrial strain parameters. Similarly, we previously reported that three CMR-derived variables reflecting diastolic function—early peak filling rate, atrial peak filling rate (APFR), and peak filling rate ratio (PFRR)—remained stable post-race in LGE + triathletes, whereas higher APFR and reduced PFRR were observed in LGE − triathletes [16]. Further, we previously reported on increased extracellular volume in the subgroup of LGE + triathletes, which might be indicative of increased myocardial stiffness [14, 16]. Thus, a possible explanation for the missing alterations between baseline and post-race biventricular and LA strain in LGE + male triathletes might be an increased myocardial stiffness.

### Study limitations

Our study has several limitations. First, this study had a relatively small sample size, which might increase the potential of type I and II errors. Second, the subjects enrolled in the present study were predominantly male triathletes. Currently, most of our knowledge on race-induced myocardial injury is also based on male athletes, due to the paucity of data on female subjects. Further studies are needed to close this gap of knowledge. Third, the study ruled out triathletes with pre-existing cardiovascular and systemic diseases, limiting its generalizability. Finally, compressed sensing might alternate strain values; subsequent studies should clarify whether strain values would be dependent on sense factors.

### Clinical implications

In this study, an endurance race led to acute alterations of biventricular and LA strain, which might be one of the mechanisms leading to an athletic cardiac remodeling. Also, it can be assumed that chronic athletic activity might be associated with an increased incidence of LGE, a potential substrate for cardiac arrhythmia. Subsequent studies should longitudinally monitor the persistence of post-race strain alterations and correlate with possible major adverse cardiac events.

## Conclusions

This prospective study analyzed the post-race changes in biventricular and biatrial myocardial strain in competitive male and female triathletes. Although there were no differences between baseline and post-race LVEF and RVEF, the alterations of biventricular and left atrial strain parameters might be a possible intrinsic compensatory mechanism following an acute bout of endurance exercise rather than myocardial dysfunction. Furthermore, there were no alterations between baseline and post-race biventricular and biatrial strain parameters in male triathletes with focal myocardial fibrosis (LGE +), which might be due to increased myocardial stiffness. The combined use of strain parameters may allow a better characterization of ventricular and atrial function at rest and post-exercise as well as the cardiac adaptation mechanisms in endurance athletes.

## Supplementary Information

Below is the link to the electronic supplementary material.

Supplementary file1 (DOCX 89 KB)

## Abbreviations

CMR: Cardiovascular magnetic resonance
ECV: Extracellular volume
EF: Ejection fraction
FT-CMR: Feature-tracking cardiac magnetic resonance
FW: Free wall
GCS: Global circumferential strain
GLS: Global longitudinal strain
GRS: Global radial strain
LA: Left atrial
LGE: Late gadolinium enhancement
LV: Left ventricle
MOLLI: Modified look-locker inversion recovery
NT-proBNP: N-terminal pro-brain natriuretic peptide
RA: Right atrial
RV: Right ventricle
STE: Speckle tracking echocardiography

## Declarations

### Guarantor

The scientific guarantor of this publication is Enver Tahir, MD.

### Statistics and biometry

No complex statistical methods were necessary for this paper.

### Study subjects or cohorts overlap

CMR findings including baseline exercise test parameters, ventricular volumes, T1 relaxation times, and distribution of focal myocardial fibrosis visualized by late gadolinium enhancement (LGE) imaging of male and female triathletes as well as LV strain values of male triathletes were reported in two previous publications. A third follow-up publication on 30 male triathletes described post-race ventricular and atrial volumes, diastolic LV filling patterns, and T1/T2 relaxation times.

The current study expands the number of triathletes by *N* = 20. Apart from *N* = 10 additional male triathletes (one with focal myocardial fibrosis), the current study also includes *N* = 10 female triathletes.

The current study focuses on the alterations of left ventricular, right ventricular, and left atrial strain in male and female triathletes as well as in athletes with focal myocardial fibrosis (LGE +) following an endurance race.

### Methodology

• prospective

• observational

• performed at one institution
