# Supplementary material for: Acute impact of an endurance race on biventricular and biatrial myocardial strain in competitive male and female triathletes evaluated by feature-tracking CMR
Source: Eur Radiol. 2021 Dec 13;32(5):3423–35. doi: 10.1007/s00330-021-08401-y (PMC9038901; doi:10.1007/s00330-021-08401-y)
Supplement: Supplementary file 1 — Supplementary file1 (DOCX 89 KB) [file 330_2021_8401_MOESM1_ESM.docx]

**Supplementary Material**

**Previously reported data**

The initial publication reported on 54 male and 29 female triathletes and included baseline exercise test parameters, ventricular volumes, T1 relaxation times, and incidence and distribution of focal LV myocardial fibrosis visualized by late gadolinium enhancement (LGE) imaging [1]. Nine of the male triathletes (17%) had focal myocardial fibrosis (LGE+) [1]. A follow-up publication reported on an expanded cohort of 78 male triathletes (19% LGE+), and the impact of focal myocardial fibrosis on global and segmental left ventricular strain [2]. A third publication focused on post-race changes in CMR parameters including ventricular and atrial volumes, diastolic LV filling patterns and T1/T2 relaxation times in thirty male triathletes only (ten LGE+) [3]. The current publication expands the post-race cohort by nine male and eleven female triathletes, including eleven LGE+ male triathletes, and focuses on post-race changes of biventricular and biatrial myocardial strain generated with the novel feature-tracking CMR technique.

**Methods and Materials**

*Endurance race details*

23 triathletes completed an Olympic distance triathlon (1.5 km swimming, 40 km cycling and 10 km running) for a cumulative time of 2.5±0.3 hours. 8 triathletes completed a marathon (42.195 km) for a cumulative time of 3.7±0.8 hours. 7 triathletes completed a sprint distance triathlon (0.5 km swimming, 20 km cycling and 5 km running) for a cumulative time of 1.3±0.2 hours. 4 triathletes completed a running competition (20.1±7.8 km) for a cumulative time of 1.7±0.8 hours. 3 triathletes completed a cycling competition (252±142 km) for a cumulative time of 9.3 ±5.5 hours. 2 triathletes completed a middle triathlon distance (2 km swimming, 80 km cycling and 20 km running) for a cumulative time of 4.7±0.1 hours. 2 triathletes completed a half marathon (21.098 km) for a cumulative time of 1.98±0.04 hours. 1 triathlete completed a duathlon (145 km cycling and 14 km running) for a cumulative time of 4.7±0.1 hours. There were no differences between the subgroups of LGE-, LGE+ and female triathletes regarding cumulative race distance (*P*=0.201), swimming distance (*P*=0.276), cycling distance (*P*=0.292), running distance (*P*=0.406) and race completion times (*P*=0.246).

*CMR protocol*

CMR was performed using a 1.5 T MR scanner equipped with a phased array surface receive coil (Achieva, Philips Healthcare).

*Cine CMR – steady-state free-precession sequence*

The CMR protocol included standard steady-state free-precession cine CMR in short axis for LV and right ventricle (RV) volumetry and LV mass with the following typical imaging parameters: acquired voxel size (AVS) 1.98 x 1.80 x 6 mm^3^, reconstructed voxel size (RVS) 1.36 x 1.36 x 6 mm^3^, gap 4 mm, 9-10 slices for full LV coverage, echo time = 1.67 ms, time to repetition = 3.34 ms, flip angle = 60°, sense factor, 2.0; 25 phases per RR interval.

*T1 Mapping – MOLLI sequence*

T1 mapping was performed using a Modified Look Locker Inversion Recovery (MOLLI) sequence with a 5s(3s)3s scheme on three short-axes slices (apical, mid and basal) before and 15 minutes after contrast-media administration as described before [1]. Typical imaging parameters were as follows: 1.97 x 2.00 x 10 mm^3^, RVS 1.17 x 1.17 x 10 mm^3^, 3 slices, echo time = 1.59 ms, time to repetition = 3.17 ms, flip angle = 35°, SENSE factor = 2, linear phase encoding, ten start-up cycles to approach steady-state prior to imaging, effective inversion times between 188 and 3382 ms.

*T2-mapping – GraSE sequence*

T2-mapping was performed before administration of contrast-media on identical three end-diastolic LV short-axes, corresponding to the MOLLI slices using a gradient- (echo planar imaging) and spin-echo multi-echo sequence (GraSE) in three short-axis sections [4]*.* Typical imaging parameters of the GraSE sequence were: Voxel size 1.05 x 1.05 x 10 mm^3^, 3 slices, nine echoes with effective echo times between 12.5 and 62.4 ms, time to repetition = 800 ms (1 RR interval), one breath-hold per slice.

*LGE imaging – phase-sensitive inversion recovery – PSIR sequence*

Ten minutes after bolus injection of 0.2 mmol/kg gadoter acid (Dotarem®, Guerbet) at a rate of 2.5 ml/s end-diastolic LGE images were acquired using end-diastolic phase-sensitive inversion recovery (PSIR) sequences: AVS 1.59 x 1.71 x 8 mm^3^, RVS 0.97 x 0.98 x 8 mm^3^, gap 2 mm, 9-10 slices, echo time = 2.40 ms, time to repetition = 5.50 ms, flip angle = 15°. The optimal inversion delay was obtained from a Look-Locker experiment. LGE images were acquired in short-axis orientation covering the entire heart and in two-, three- and four-chamber views.

*CMR data analysis*

Two investigators (H.C. and M.W. with 3 and 4 years of experience in CMR, respectively) independently and blindly analyzed each CMR using a commercially available software (CVi42, Circle Cardiovascular Imaging Inc.). CMR parameters were indexed to the calculated body surface area (BSA) and are given as the mean of the two observers. Evaluation of LV and RV volumes and LV mass was performed in standard fashion on the short-axis cine stack [5]. The epicardial and endocardial borders of the myocardium were manually traced on end-diastolic and end-systolic images at each anatomic level encompassing the entire heart from apex to base. Trabeculae and papillary muscles were included in the ventricular volumes and excluded from the myocardial mass for reproducibility [6]. LV mass was calculated by multiplying the myocardial volume by the specific weight of cardiac muscle (1.05 mg/mL) [5]. Left (LA) and right atrial (RA) volumes and were quantified using the biplane area-length method, excluding pulmonary veins and atrial appendage [7]. Presence of LGE was analyzed visually [5]. Native T1, post-contrast T1 and ECV maps were generated using a dedicated plug-in written for the OsiriX™ software [8]. Native T1, post contrast T1 and ECV were obtained by averaging measures from basal and midventricular short-axis slices to yield final measurements [9]. Meticulous care was taken not to include the blood volume into the measurements to avoid partial volume effects [9]. Areas of focal LGE were excluded from T1 and ECV measurements to evaluate these parameters unbiased from presence of LGE. ECV was calculated using the previously established equation [10-13].

ECV = [1-hematocrit]*[ΔR1]*_myocardium_*/[ΔR1]*_blood pool_*

In this equation, R1 is defined as 1/T1 and Δ as the difference between pre- and late post-contrast R1 values [10-13]. Hematocrit was measured from a venous blood sample taken on the same day of CMR scan.

**Data Supplement References**

1 Tahir E, Starekova J, Muellerleile K et al (2018) Myocardial Fibrosis in Competitive Triathletes Detected by Contrast-Enhanced CMR Correlates With Exercise-Induced Hypertension and Competition History. JACC Cardiovasc Imaging 11:1260-1270

2 Tahir E, Starekova J, Muellerleile K et al (2019) Impact of Myocardial Fibrosis on Left Ventricular Function Evaluated by Feature-Tracking Myocardial Strain Cardiac Magnetic Resonance in Competitive Male Triathletes With Normal Ejection Fraction. Circ J 83:1553-1562

3 Tahir E, Scherz B, Starekova J et al (2020) Acute impact of an endurance race on cardiac function and biomarkers of myocardial injury in triathletes with and without myocardial fibrosis. Eur J Prev Cardiol 27:94-104

4 Baessler B, Schaarschmidt F, Stehning C, Schnackenburg B, Maintz D, Bunck AC (2015) Cardiac T2-mapping using a fast gradient echo spin echo sequence - first in vitro and in vivo experience. J Cardiovasc Magn Reson 17:67

5 Schulz-Menger J, Bluemke DA, Bremerich J et al (2020) Standardized image interpretation and post-processing in cardiovascular magnetic resonance - 2020 update : Society for Cardiovascular Magnetic Resonance (SCMR): Board of Trustees Task Force on Standardized Post-Processing. J Cardiovasc Magn Reson 22:19

6 Papavassiliu T, Kuhl HP, Schroder M et al (2005) Effect of endocardial trabeculae on left ventricular measurements and measurement reproducibility at cardiovascular MR imaging. Radiology 236:57-64

7 Le TT, Tan RS, De Deyn M et al (2016) Cardiovascular magnetic resonance reference ranges for the heart and aorta in Chinese at 3T. J Cardiovasc Magn Reson 18:21

8 Radunski UK, Lund GK, Stehning C et al (2014) CMR in patients with severe myocarditis: diagnostic value of quantitative tissue markers including extracellular volume imaging. JACC Cardiovasc Imaging 7:667-675

9 Schelbert EB, Piehler KM, Zareba KM et al (2015) Myocardial Fibrosis Quantified by Extracellular Volume Is Associated With Subsequent Hospitalization for Heart Failure, Death, or Both Across the Spectrum of Ejection Fraction and Heart Failure Stage. J Am Heart Assoc 4

10 Ugander M, Oki AJ, Hsu LY et al (2012) Extracellular volume imaging by magnetic resonance imaging provides insights into overt and sub-clinical myocardial pathology. Eur Heart J 33:1268-1278

11 Arheden H, Saeed M, Higgins CB et al (1999) Measurement of the distribution volume of gadopentetate dimeglumine at echo-planar MR imaging to quantify myocardial infarction: comparison with 99mTc-DTPA autoradiography in rats. Radiology 211:698-708

12 Schelbert EB, Testa SM, Meier CG et al (2011) Myocardial extravascular extracellular volume fraction measurement by gadolinium cardiovascular magnetic resonance in humans: slow infusion versus bolus. J Cardiovasc Magn Reson 13:16

13 Kellman P, Wilson JR, Xue H et al (2012) Extracellular volume fraction mapping in the myocardium, part 2: initial clinical experience. J Cardiovasc Magn Reson 14:64

**Supplemental Table 1:** Demographics and CMR parameters of LGE- triathletes at baseline and post-race.

|  | **Baseline**  LGE- (n= 29) | **Post-race**  LGE- (n= 29) | ***p* value** |
| --- | --- | --- | --- |
| **Clinical parameters** |  |  |  |
| Body surface area, m² | 2.03 ±0.13 | 2.01 ±0.13 | <0.05 |
| Troponin T, pg/ml | 6 ±3 | 54 ±87 | <0.01 |
| NT-proBNP, pg/ml | 30 ±21 | 100 ±58 | <0.0001 |
| CK, U/l | 237 ±172 | 547 ±305 | <0.0001 |
| CK-MB, U/l | 14 ±16 | 34 ±17 | <0.0001 |
| Systolic BP at rest, mmHg | 125 ±11 | - | - |
| Diastolic BP at rest, mmHg | 85 ±9 | - | - |
| **CMR – left heart** |  |  |  |
| Heart rate, bpm | 53 ±8 | 69 ±10 | <0.0001 |
| LV cardiac index, l/min/m^2^ | 3.26 ±0.59 | 3.94 ±0.61 | <0.0001 |
| LVEF, % | 60 ±5 | 61 ±7 | 0.434 |
| LV mass index, g/m² | 77 ±10 | 78 ±11 | 0.228 |
| LVEDVi, ml/m² | 102 ±16 | 95 ±15 | <0.01 |
| LVESVi, ml/m² | 41 ±9 | 37 ±11 | <0.05 |
| LVSVi, ml/m² | 61 ±10 | 58 ±9 | 0.113 |
| LAEDVi, ml/m² | 19 ±9 | 15 ±6 | <0.01 |
| LAESVi, ml/m² | 46 ±12 | 34 ±9 | <0.0001 |
| **CMR – right heart** |  |  |  |
| RVEF, % | 57 ±8 | 58 ±8 | 0.317 |
| RVEDVi, ml/m² | 104 ±18 | 101 ±19 | 0.302 |
| RVESVi, ml/m² | 46 ±14 | 44 ±14 | 0.072 |
| RVSVi, ml/m² | 58 ±9 | 57 ±9 | 0.864 |
| RAEDVi, ml/m² | 28 ±9 | 28 ±11 | 0.879 |
| RAESVi, ml/ m² | 51 ±13 | 46 ±11 | <0.01 |
| **CMR – Strain** |  |  |  |
| LV GLS, % | -17 ±2 | -16 ±2 | <0.05 |
| LV GCS, % | -15 ±3 | -16 ±3 | <0.01 |
| LV GRS, % | 36 ±8 | 41 ±9 | <0.05 |
| RV GLS, % | -19 ±3 | -20 ±5 | 0.562 |
| RV GCS, % | -7 ±2 | -7 ±3 | 0.068 |
| RV FW, % | -20 ±6 | -20 ±8 | 0.644 |
| LA GLS, % | 29 ±7 | 23 ±6 | <0.001 |
| RA GLS, % | 24 ±5 | 23 ±6 | 0.344 |
| **CMR – Mapping** |  |  |  |
| Native T1, ms | 978 ±21 | 985 ±19 | 0.229 |
| Native T2, ms | 53 ±2 | 52 ±4 | 0.394 |
| ECV, % | 24.9 ±1.5 | - | - |

Numbers are mean ±SD for continuous and n (%) for categorical data.

Baseline and post-race data were partly reported in previous publications as indicated in the materials and methods section.

**Abbreviations:** as in Table 1.
